# Dr. Melchiorre Guzzoni

**Published:** 1885-04

**Authors:** 


### OBITUARY.

### DR. MELCHIORRE GUZZONI.

Dr. Melchiorre Guzzoni, Professor of Pathology and Director of the Clinic at the Milan Veterinary School, died suddenly on the evening of February 17th, aged 40 years. His death is deplored as an irreparable loss by his colleagues, and by the pupils of the school, where he was held in the highest esteem, and considered an eminent scholar and a successful teacher, and by the profession at large, in which he was numbered as one of its most brilliant members.

